# Musical Distractions: Music-Based Rhythmic Auditory Stimulation Fails to Improve Gait in Huntington’s Disease

**DOI:** 10.3390/brainsci15080820

**Published:** 2025-07-31

**Authors:** Sidney T. Baudendistel, Lauren E. Tueth, Allison M. Haussler, Gammon M. Earhart

**Affiliations:** 1 Program in Physical Therapy, Washington University in St. Louis School of Medicine, St. Louis, MO 63110, USAlaurentueth@gmail.com (L.E.T.);; 2 Department of Neurology, Washington University in St. Louis School of Medicine, St. Louis, MO 63110, USA; 3 Department of Neuroscience, Washington University in St. Louis School of Medicine, St. Louis, MO 63110, USA

**Keywords:** Huntington’s disease, gait, dual task, cueing, rhythmic auditory stimulation, cognitive performance, balance confidence

## Abstract

Background/Objectives: Huntington’s disease (HD) is a neurodegenerative disorder involving the basal ganglia and is characterized by psychiatric, cognitive, and movement dysfunction, including gait and balance impairment. Given the limited efficacy of pharmacological treatments for HD motor symptoms, nonpharmacological approaches like rhythmic auditory stimulation are being explored. This study aims to describe walking performance in people with HD during rhythmic auditory stimulation using external musical cues and internal singing cues. Methods: Individuals in the manifest stage of HD performed walking in four conditions: (1) comfortable pace, (2) cognitive dual task, (3) musical cue (music was played aloud), and (4) singing cue (participants sang aloud). Sensors measured cadence, velocity, stride length, and variability. Relationships between change in cadence and motor and cognitive measures were explored. Results: While no direct measurements of synchronization were performed, limiting our interpretation, neither the external musical cue nor the singing cue significantly improved walking performance. Both cues increased variability, similar to what was observed during the dual task. Greater subjective balance confidence and better cognitive performance were associated with positive cadence change during cueing. Conclusions: Musical cues may be too cognitively demanding for individuals with Huntington’s disease as they worsen gait variability without increasing gait speed, cadence, or stride length. Although global cognition and perceived balance confidence were related to the ability to increase cadence, very few people were able to increase their cadence during either cue. Therefore, the results do not support the use of musical cues to improve gait for individuals with Huntington’s disease.

## 1. Introduction

Huntington’s disease (HD) is an inherited movement disorder affecting 3.92 people per 100,000 worldwide [1]. In the US alone, 41,000 individuals are symptomatic, but more than 200,000 are at risk of inheriting the disease [2]. While the condition is considered a neuropsychiatric disorder characterized by behavioral, psychiatric, and cognitive dysfunction, movement dysfunction, in the form of dystonic and hyperkinetic movements (i.e., chorea), is also common [3]. Also, individuals with HD often display bradykinesia of the limbs [3]. While bradykinetic features may not be as outwardly apparent as hyperkinetic movements, bradykinetic features have been shown to be more debilitating for individuals with HD than chorea and may serve as a better marker for disease progression [4,5]. Indeed, bradykinesia may be a contributing factor to gait impairment and loss of mobility, which are apparent throughout the disease, including in individuals in the premanifest stage [6,7,8,9]. Compared to individuals without HD, individuals with HD walk significantly slower, with reduced cadence and shorter stride length [10]. These aspects of gait worsen with disease progression [10]. Additionally, variability in stride time and stride length is increased in individuals with HD compared to controls [10] and is related to both disease severity [11,12] and fall risk [13,14]. Gait variability is higher in HD than in other conditions, such as Alzheimer’s disease, cerebellar ataxia, and Parkinson’s disease (PD) [15]. As HD progresses, difficulties with walking contribute significantly to disability and negatively impact overall quality of life [10].

Unfortunately, there are no disease-modifying drugs for HD, and pharmacological treatments for the motor symptoms of HD are limited [16,17,18]. According to a review of treatments for HD, there are no pharmacological treatments available that are likely to improve gait ability [18]. While certain medications may improve motor symptoms such as chorea, they can also exacerbate other symptoms, including mood and cognition [19]. Given the limited availability of pharmacological treatments that specifically target motor symptoms, nonpharmacological approaches such as physical therapy and rehabilitation have gained increasing importance [16,20]. According to clinical recommendations for physical therapy for individuals with HD [20], supervised gait training is recommended with “grade A evidence” to improve spatiotemporal features of gait. One strategy that has been investigated is rhythmic auditory stimulation (RAS), in which patients are asked to match their footfalls to rhythmic cues, often presented as a metronome or with music [21]. RAS is well-established to improve walking in other movement disorders that present with high gait variability, such as PD [22,23,24,25]. In contrast to the repeatability of cueing benefits reported in those with PD, there are mixed results for those with HD [26,27,28,29]. For example, Thaut et al. found that a metronome set to a tempo faster than normal cadence significantly increased cadence, gait speed, and stride length in people with HD [26]. Delval et al. were not able to confirm the findings of statistical improvement in gait speed with the use of cues from a metronome but noted there was a “trend toward improvement”, including a reduction in gait variability [27]. Across studies, authors note a clear impairment in the ability to synchronize each step to the cue, often hypothesized to be due to deficits in cognition [26,27,28,29]. For individuals with HD, RAS may be similar to a cognitive dual task, reducing gait performance instead of improving it. However, direct comparisons between cueing and dual tasking in people with HD have not been completed.

Despite the mixed results across studies, RAS is still suggested as a treatment for gait issues in HD [30]. This is partly due to its relative safety and ease of clinical implementation, as RAS requires no specialized equipment. Furthermore, the use of art-based therapies, including music-based RAS, in HD is limited but promising [31]. For individuals with PD, musical cueing, in which a rhythmic song is played, may be more beneficial than a simple metronome [32,33,34]. Work from our laboratory has further demonstrated that singing may specifically target reductions in gait variability, as singing focuses on internal generation of rhythm instead of response to an external cue [35]. To our knowledge, only one study investigated the use of music-based cueing in people with HD [26]. Thaut et al. found participants were able to increase velocity, stride length, and cadence when walking to “the instrumental version of a folk song”, although none of these increases were significant [26]. Compared to metronome cueing, the music-based cue was less effective [26]. While no cognitive measures were collected, the authors hypothesized that reduced cognition was a contributing factor to the lack of synchronization to music-based cues in this population.

While RAS is well-researched in other populations, this study is novel in three ways: (1) no previous studies directly compared cueing and dual-task walking in HD, (2) no previous studies investigated the use of internally generated singing cues in those with HD, and (3) no previous studies statistically investigated the effect of cues on gait variability in individuals with HD. The primary purpose of the current study is to compare walking tasks in people with HD, specifically uncued walking at a comfortable pace, walking with a cognitive task, walking with external musical cueing, and walking with internally generated singing aloud cueing. We hypothesize that there will be a significant difference between the four walking tasks, with gait performance worsening during dual tasking and improving with the singing cue relative to uncued walking, whereas the external musical cue will have little impact on gait. We also conducted an exploratory analysis to investigate the relationships between motor and global cognitive impairments and the ability to increase cadence with cueing in this population. Similar to Thaut et al. [26], we hypothesize that greater motor and cognitive dysfunction will be associated with reduced ability to alter gait during either form of cueing.

## 2. Materials and Methods

Participants were part of a larger cross-sectional and observational study investigating mobility in individuals with Huntington’s disease [36]. This study was funded by the Program in Physical Therapy at Washington University in St. Louis School of Medicine and was approved by the Institutional Review Board. The analyses herein represent new and previously unpublished work.

### 2.1. Participants

Participants were recruited from the Movement Disorders Clinic at Washington University School of Medicine. The inclusion criteria for the larger study included being in the manifest stage of gene-positive HD and self-reported ability to walk for ten continuous minutes without assistance from another person. Participants with conditions that may make safe participation difficult, such as other neurological, cardiovascular, or orthopedic conditions, were excluded. All participants completed one in-person visit.

### 2.2. Protocol

As part of the larger protocol [36], all participants self-reported demographic information, including past medical history, current medication regimen, and 1-month fall history. Next, participants completed the Montreal Cognitive Assessment (Version 8.1) (MoCA) [37], where scores of 25 or less (out of 30) indicated impairment in HD [38]. Education level was adjusted for, as recommended. Cognitive impairment was not exclusionary. For motor testing, including balance and gait tasks, participants wore a gait belt and were shadowed by a study staff member to ensure safety. Participants’ motor function was assessed by a certified rater using the Unified Huntington’s Disease Rating Scale Total Motor Score (UHDRS-TMS), where items are scored on a scale of 0 to 4, with a total possible score of 124. Higher scores indicate greater motor impairment [39]. Balance was assessed using the Balance Evaluation Systems Test (BESTest) [40]. The BESTest includes 36 items, scored on a scale of 0–3, with a total possible score of 108. Higher scores indicate better balance.

Following the balance assessment, all participants underwent a series of gait tasks. All gait tasks were completed in a 100-foot (~30 m) hallway. Participants were outfitted with six wearable inertial measurement sensors (APDM Opal V2R, Clario, Portland, OR, USA), placed on both feet, both wrists, the sternum, and the lumbar spine [41]. In addition to the comfortable pace walking and cognitive dual-task walking reported in Tueth et al. [36], two additional tasks were completed to investigate the effect of musical cueing in individuals with HD. Similar to previous studies from our laboratory [35,42], two cueing types were tested: (1) an externally based cue of music being played aloud (MUSIC) and (2) internally generated cueing of participants singing aloud (SING). For all cued tasks, a piano version of “Skip to my Lou” was used, as it is readily known and can be easily taught to those unfamiliar with the song. A well-known song was selected as familiarity may be related to the benefits of musical cueing [43]. To test basic familiarity with the lyrics, participants sang a round of the song while seated. All participants were able to complete this task. For walking trials, the musical cue was played via speakers and could be heard by the participant at all points in the hallway. The cue rate was set to 110% of the preferred cadence, rounded to the nearest 5 bpm. While Thaut et al. aimed to test participants at 120% of baseline cadence to focus on improving velocity, not all participants in their study were able to achieve this step rate [26]. As all participants in the Thaut et al. study were able to achieve at least 110% [26], we set the cadence for all participants in the present study to 110%. Slower tempos of 80 bpm [28,29] or 90% of baseline cadence have been tested in previous studies in HD, resulting in slower velocity and shorter stride length [26,29]. Therefore, slower-than-baseline tempos were not considered. Additionally, 110% was chosen as it has been shown to be more beneficial for reducing gait variability compared to 120% in people with PD [44]. Trial order was not randomized, similar to Thaut et al. [26]. As this study was considered exploratory, the MUSIC trials always came prior to the SING trials to facilitate learning of the tasks and to increase task complexity gradually [43].

After SING trials, an additional round of uncued walking was completed at the end of the other gait tasks. As the set trial order could introduce order or fatigue effects, these additional uncued trials were compared to the initial uncued walking task to explore potential carryover of cueing or potential fatigue, similar to Thaut et al. [26].

Participants completed three 30 s trials in each walking task. If needed, up to two additional trials were performed when participants appeared to misunderstand instructions, when instrumentation malfunctioned, or due to environmental disruptions (e.g., someone inadvertently walking into the hallway and disrupting a trial).

The trial order and information are as follows:

1. Pre Uncued (PRE): The first uncued walking trials were used as the baseline value for all participants. The cadence of steady-state walking, as determined by APDM Mobility Lab software, for each trial was averaged together and multiplied by 1.10 to represent each participant’s individualized cueing tempo for MUSIC and SING tasks.
  - Instructions: “…walk at your comfortable pace”.
2. Cognitive Dual Task (DT): Direct comparison between baseline walking and DT can be found in [36]. For this study, the verbal fluency cognitive dual task was included as a comparison to the cueing paradigms.
  - Instructions: “Name as many words as you can that start with a specific letter while you walk”. No specific instruction for the prioritization of either task was provided.
3. 110% Musical Cue (MUSIC): Participants listened to one round of the song at their individualized tempo. After an auditory signal to begin walking, the same song continued to loop continuously for 30 s.
  - Instructions: “After one verse, the music will keep playing and you can begin walking. Keep walking on the beat until the music stops”.
4. 110% Singing Cue (SING): Similar to MUSIC, participants listened to one round of the song at their individualized tempo. After the auditory signal to begin walking, the music stopped, and participants were asked to begin singing aloud and walking to the beat of their own singing, trying to match the same tempo they had just heard. No specific instructions for prioritization of either singing or walking were provided.
  - Instructions: “When the music stops, start singing the song and walking to the beat. Keep walking and singing until the tone sounds”.
5. Post Uncued (POST): One final set of three trials was conducted to investigate the effects of the cueing on comfortable pace walking, as results may assist in understanding the role that fatigue may play in this population.
  - Instructions: “…you will again walk at your comfortable walking pace”.

We did not directly measure synchronization of footfall timing compared to the auditory beat, but rather focused on gait performance during the cue, similar to our past work in people with PD [35,44]. For measures of gait, our primary outcome was cadence. Secondary outcomes included velocity, stride length, and variability in step time and stride length, calculated as the coefficient of variation (CV) [45].

Following the gait tasks, all participants completed additional questionnaires, including the Activities-Specific Balance Confidence Scale (ABC). The ABC provides a “wide spectrum of activity difficulty” to investigate which activities of daily living may cause fearfulness in participants [46]. The ABC is a 16-item scale scored from 0 to 100, in which 0% represents no confidence and 100% is complete confidence. In general, lower balance confidence is associated with greater fall risk [47].

### 2.3. Statistical Analysis

Data were checked for normality using Kolmogorov–Smirnov tests. Due to the interdependence of gait variables, a repeated measures MANOVA (multivariate analysis of variance) was used to analyze the potential effects of task condition on gait. Wilks’ Lambda was reported for the MANOVA. If indicated, one-way repeated measures ANOVAs were used to analyze the potential differences for each individual gait variable between PRE, DT, MUSIC, and SING. Sphericity was tested with Mauchly’s Test and, if significant, Greenhouse–Geisser corrections were used. Pairwise comparisons between PRE and DT trials were reported for stride length, velocity, and double support percent in Tueth et al. [36]. The differences between PRE and DT for gait measures were not the focus of this study but are reported for completeness. Planned comparisons were run to compare each measure between PRE, MUSIC, and SING; *p*-values were set to *p* < 0.05 with Bonferroni adjustment for multiple comparisons during pairwise comparisons. Furthermore, planned comparisons were run to compare each measure between DT, MUSIC, and SING; *p*-values were set to *p* < 0.05 with Bonferroni adjustment for multiple comparisons during pairwise comparisons.

Separately, paired *t*-tests for all gait variables were run between PRE and POST to investigate the potential carryover effects or effects of fatigue; *p*-values were set to *p* < 0.05 with Bonferroni adjustment.

For the exploratory hypothesis, measures of motor and cognitive impairment were correlated to a change in cadence during both MUSIC and SING trials. Specifically, to explore the relationship between motor and cognitive impairment and the ability to increase cadence, Pearson correlations were calculated between the cadence change in cueing trials and four measures of interest: the MoCA, the UHDRS-TMS, the BESTest, and ABC. Specifically, cadence change was calculated as the percent change between PRE and the individual cueing tasks. A value of 0% indicates no change from PRE, and values greater than 0% represent an increased cadence compared to PRE. As this was an exploratory hypothesis, statistical significance was set at *p* < 0.05.

## 3. Results

Twenty-two individuals with HD were included in these analyses (Table 1). Individuals were taking the following medications related to their HD symptoms: vesicular monoamine transporter 2 (VMAT) inhibitor (*n* = 2), benzodiazepine (*n* = 5), antipsychotic medication (*n* = 6), tetracyclic antidepressant (*n* = 6), serotonin-norepinephrine reuptake inhibitor (*n* = 3), selective serotonin reuptake inhibitor (*n* = 12), and anticonvulsant for mood stabilization (*n* = 4). Participant-specific demographics and medication status are available in Appendix A. Variability in stride length and variability in stride time were not normally distributed according to Kolmogorov–Smirnov tests. Therefore, variability measures were log-transformed, similar to Lord et al. [48]. After transformation, these measures were considered normal. Means for variability measures are reported as untransformed variables for clarity. Log-transformed means are reported in Appendix B.

**Figure 1:**
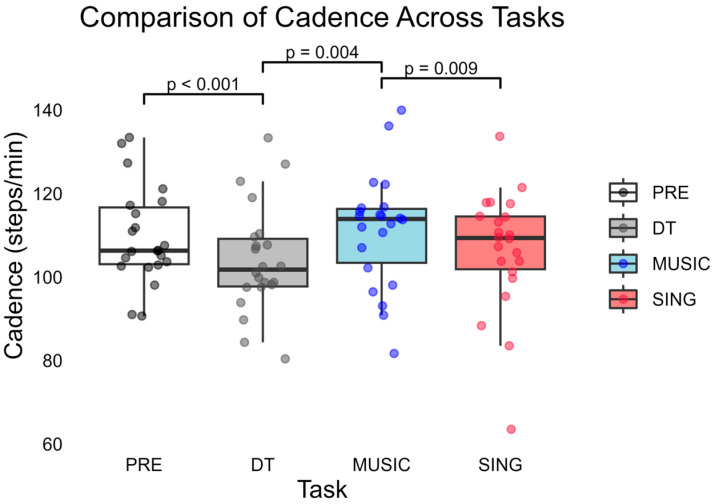
Comparison of mean cadence across tasks. Only significant *p*-values are reported in the figure; for all the others, *p* > 0.05. Abbreviations: baseline, uncued walking trials (PRE); cognitive dual-task trials (DT); externally based cue of music being played aloud (MUSIC); and internally generated cueing of participants singing aloud (SING).

At the multivariate level, there was a significant effect of task (Wilks’ Λ = 0.058, F(15,7) = 7.643, *p* = 0.006). One-way repeated measures ANOVA revealed a significant effect of task on the primary measure, i.e., cadence (F(1.791) = 6.051, *p* = 0.007; Figure 1).

**Figure 2:**
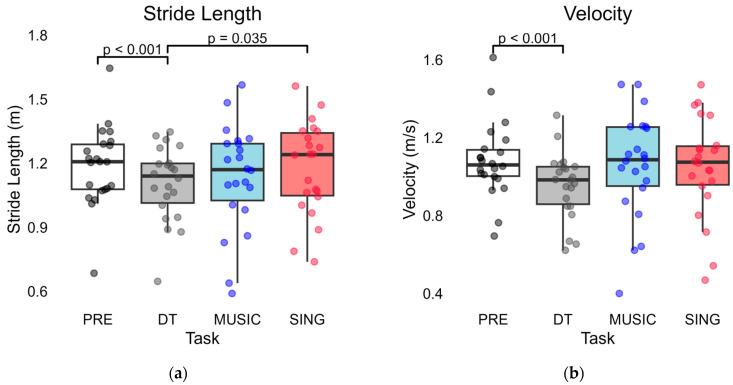

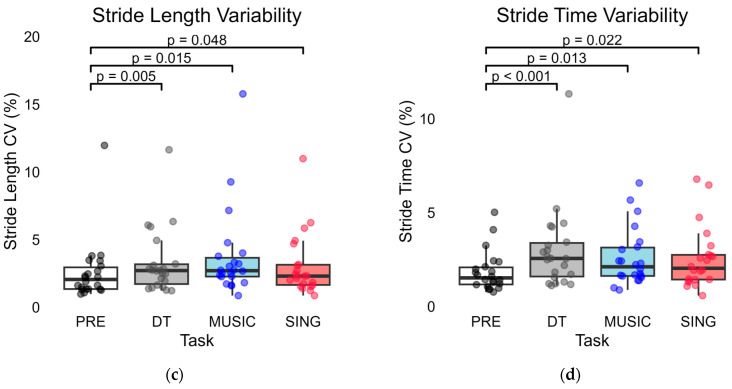
Comparison of secondary gait outcomes across tasks. Only significant *p*-values are reported in the figure; for all the others, *p* > 0.05. Abbreviations: baseline, uncued walking trials (PRE); cognitive dual-task trials (DT); externally based cue of music being played aloud (MUSIC); and internally generated cue of participants singing aloud (SING). (**a**) Comparison of stride length across tasks. (**b**) Comparison of velocity across tasks. (**c**) Comparison of stride length variability modeled as a coefficient of variation (CV) across tasks. (**d**) Comparison of stride time variability modeled as a coefficient of variation (CV) across tasks.

There was also a significant effect of task on all secondary measures of gait, including stride length (F(1.56) = 3.991, *p* = 0.037; Figure 2a), velocity (F(1.594) = 4.925, *p* = 0.019; Figure 2b), stride length variability (F(2.029) = 6.319, *p* = 0.004; Figure 2c), and stride time variability (F(3) = 9.062, *p* < 0.001; Figure 2d). Means and standard deviations are shown in Table 2.

### 3.1. PRE Compared to DT Trials

Compared to PRE, participants with HD walked with reduced cadence (*p* = 0.001), smaller stride length (*p* < 0.001), and reduced velocity (*p* < 0.001) in the DT task, as previously reported by Tueth et al. [36]. DT trials had increased stride length variability (*p* = 0.005) and stride time variability (*p* < 0.001) relative to PRE.

### 3.2. PRE Compared to MUSIC and SING Trials

For cadence, stride length, and velocity, there were no significant differences between PRE and MUSIC (*p* = 1.000, *p* = 0.973, *p* = 1.000, respectively) or between PRE and SING (*p* = 1.000, *p* = 1.000, *p* = 1.000, respectively).

For stride length CV, there was a significant difference after Bonferroni correction between PRE and MUSIC (*p* = 0.015) and between PRE and SING (*p* = 0.048). Participants demonstrated higher stride length variability in both MUSIC and SING trials relative to PRE.

For stride time CV, there was a significant difference between PRE and MUSIC trials (*p* = 0.013) and between PRE and SING trials (*p* = 0.022). Participants demonstrated higher stride time variability in both MUSIC and SING trials compared to PRE.

### 3.3. MUSIC Compared to SING Trials

Cadence was significantly higher in MUSIC compared to SING (*p* = 0.009), but there was no difference in stride length (*p* = 0.214), velocity (*p* = 1.000), stride length variability (*p* = 0.223), or stride time variability (*p* = 1.000) between MUSIC and SING.

### 3.4. Cue Trials Compared to DT

Both MUSIC and SING trials had greater cadence compared to DT, but only MUSIC was significantly different (*p* = 0.004); SING was not significantly different (*p* = 1.000). MUSIC and SING trials had longer stride length compared to DT, but this was only significant between SING and DT (*p* = 0.035), not MUSIC and DT (*p* = 1.000). There was no significant difference in velocity between MUSIC and DT (*p* = 0.132) or between SING and DT (*p* = 0.103). This was also true for variability measures, as there was no difference between MUSIC and DT (stride length CV *p* = 1.000, stride time CV *p* = 1.000) or between SING and DT (stride length CV *p* = 1.000, stride time CV *p* = 0.820).

### 3.5. PRE Compared to POST

There were no significant differences between PRE and POST for any gait measure (*p* ≥ 0.396). Full statistical output, including means and standard deviations, can be found in Appendix C.

### 3.6. Associations with Cadence Change

One participant was unable to complete the MoCA due to the inability to write; so, the Telephone MoCA (T-MoCA) was performed in person instead. The T-MoCA is a validated assessment that excludes visual cues and drawing and can be converted to a MoCA score using an equation from Katz et al. [49]. Of note, removing this person from the dataset for the correlation analysis does not alter the significance of correlations.

There were no significant associations between cadence during PRE and MoCA (r = 0.184, *p* = 0.413), UHDRS score (r = −0.069, *p* = 0.762), BEST score (r = 0.198, *p* = 0.376), or ABC (r = 0.043, *p* = 0.851).

#### 3.6.1. Associations with Cadence Change During MUSIC

**Figure 3:**
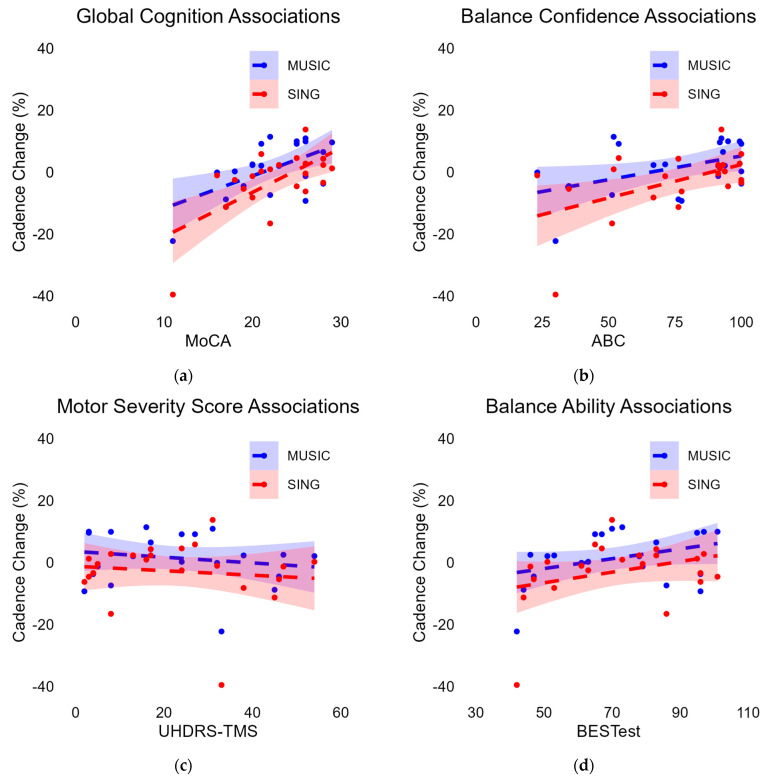
Relationships between motor and cognitive characteristics and the cadence change during the cueing, measured as a percent change from the baseline. Dots represent individual participants, dashed line is the line of best fit, and shading represents the 95% confidence interval around the line of best fit. For all panels, blue represents results from the MUSIC task, while red represents results from the SING task: (**a**) Significant correlation between the Montreal Cognitive Assessment and cadence change for MUSIC (r = 0.577, *p* = 0.005) and SING (r = 0.640, *p* = 0.001). (**b**) Significant correlation between the Activities-Specific Balance Confidence Scale and cadence change for MUSIC (r = 0.447, *p* = 0.037) and SING (r = 0.510, *p* = 0.015). (**c**) No significant correlation between the Unified Huntington’s Disease Rating Scale Total Motor Score and cadence change for MUSIC (r = −0.18, *p* = 0.424) and SING (r = −0.114, *p* = 0.612). (**d**) No significant correlation between the Balance Evaluation Systems Test and cadence change for MUSIC (r = 0.362, *p* = 0.098) and SING (r = 0.319, *p* = 0.148).

There were significant correlations (Figure 3) between cadence percent change with MUSIC and the MoCA (r = 0.577, *p* = 0.005) and the ABC (r = 0.447, *p* = 0.037). There was no significant correlation between cadence percent change and UHDRS score (r = −0.18, *p* = 0.424) nor BESTest score (r = 0.362, *p* = 0.098).

#### 3.6.2. Associations with Cadence Change During SING

Similar to the MUSIC trials, there were significant correlations (Figure 3) between cadence percent change for SING and the MoCA (r = 0.640, *p* = 0.001) and the ABC (r = 0.510, *p* = 0.015). There was no significant correlation between percent change and UHDRS score (r = −0.114, *p* = 0.612) or BESTest score (r = 0.0.319, *p* = 0.148).

## 4. Discussion

To our knowledge, this is the first study to directly compare dual-task walking performance to any cueing paradigm in HD. While cognition has often been hypothesized to be a limiting factor for individuals with HD to complete a cueing task, these associations have not been explored statistically. Our results indicate that externally played cueing and internally generated cueing do not improve walking and may worsen some aspects of gait. Specifically, participants walked with greater variability for stride length and stride time during cued tasks. While there were significant differences when comparing the RAS tasks to the DT, these differences were small. While the lack of randomization could have introduced order or fatigue effects, there was no difference in gait between PRE and POST. Finally, motor severity and objective balance ability were not associated with changes in cadence during the cueing tasks in the exploratory analysis. However, greater perceived subjective balance confidence and greater cognitive performance were associated with positive changes in cadence. Collectively, we speculate that music-based cueing may be too cognitively demanding for individuals with HD to utilize appropriately. Perceived balance confidence and global cognition may be contributing factors in performing a music-based cueing task for individuals with HD.

RAS has been proven to improve multiple aspects of gait in people with PD [25,50], driving researchers and clinicians to explore RAS in individuals with other basal ganglia disorders [51]. Degeneration of the basal ganglia is a key characteristic in both HD and PD [52,53], resulting in greater inhibition of the thalamus, reducing the speed and size of volitional movements [54,55]. These changes can be observed with a hypokinetic gait, including shorter and smaller steps [36,45,56]. Automaticity and regular control of movements are also regulated by the basal ganglia, which is reflected in the increases in gait variability in both HD and PD [9,45,56,57]. Providing a consistent auditory rhythm, including musical cueing, may help to regulate gait dysfunction by potentially bypassing the dysfunctional basal ganglia through the prefrontal cortex, auditory cortex, temporal gyri, and cerebellum [58,59,60,61,62,63,64,65]. It appears that these regions, especially the cerebellum, may compensate for poor motor functioning in both diseases [66,67,68,69]. However, in those with HD, the cerebellum atrophies early in the disease [70] and cerebellar dysfunction is associated with the onset of motor signs [71,72,73]. In PD, there is no association between cerebellar activation and disease duration [74], and hyperactivity of the cerebellum increases longitudinally [75]. In a systematic review of the role of the cerebellum in HD, the authors state that “the loss of the compensation role of the cerebellum in HD may be an explanation for the clinical onset of HD” [71]. While we did not measure brain activation, the lack of ability to utilize cues aligns with this hypothesis. The current paradigm has been shown to improve both mean spatiotemporal gait measures and gait variability in people with PD [35,42]. Here, we demonstrated that, for individuals with HD, there was no significant difference in mean values of cadence, speed, and stride length during the cueing tasks compared to comfortable pace walking, matching the results observed by past studies [27,28,29]. However, this is the first study, to our knowledge, to investigate the effect of cues on gait variability in individuals with HD statistically. Our findings of significantly larger variability during the cueing tasks compared to PRE indicate that gait instability increases during the cueing paradigm [76,77,78,79], potentially putting participants at risk for falls.

People with HD have a difficult time completing dual-task paradigms [80,81,82,83] and often show greater gait impairment during DT compared to otherwise healthy controls [80,81,83]. As reported by Tueth et al., our sample with HD walked slower, with shorter strides and longer double limb support time than older adult controls [36]. Here, we expanded these results and further assessed cadence and variability in walking during the DT trials. We found that during the DT trials, individuals with HD also walked with reduced cadence and greater stride length variability and stride time variability, aligning with other studies on dual-task walking in HD [82,84,85]. The reduction in gait performance during a cognitive dual task in HD is thought to be related to executive dysfunction [85,86], including deficits in divided attention [87], selective attention [88], and set switching [89,90]. Additionally, greater decrements in gait during a dual task in HD have been correlated to worse verbal fluency, worse scoring of the Stroop test (a measure of attention), and a lower Mattis dementia rating score [82,83]. In people with PD, auditory-based cueing actually improves walking abilities, potentially inferring that cueing is not so demanding as to be considered a dual task [91,92,93,94]. Indeed, auditory cueing strategies can be used to improve gait performance during a dual task [91,92,93,94]. For individuals with PD, MUSIC and SING trials completed at 100% of baseline walking cadence did not alter velocity, stride length, or cadence, while a verbal dual task significantly decreased all three metrics [42]. Further, the SING task improved gait variability more than the MUSIC task, and both tasks had significantly less variability than the DT, suggesting singing a familiar song may not require as many cognitive resources as a verbal dual task does in PD [42]. This is not the case for the individuals with HD in the present study, as variability during cued trials was not statistically different from DT. We speculate that the MUSIC and SING cues may be too complex for individuals with HD to incorporate. As no objective measure of cognitive load was collected during the cueing task and compared to the DT, this remains a point of interest for future studies. Importantly, our results align with similar studies, including Thaut et al., in which participants were able to alter gait with a metronome but not a music-based cue [26]. Delval et al. found that there was no significant difference in dual-task performance with or without the addition of a metronome [27], further supporting this hypothesis. Interestingly, during the MUSIC trials, cadence did increase significantly compared to DT and slightly compared to PRE; we think that this may be due to the designated purpose of each task. In the DT, participants were asked to complete two tasks simultaneously, thus splitting their attention, with no preference toward naming words or walking. This was compared to the MUSIC trial, where matching the faster beat was the specific goal. When asking individuals with HD to walk faster or “as fast as possible”, individuals increased velocity, cadence, and step length [26,28,95]. During the MUSIC trials, participants were asked to match their feet to the beat of the song, with no instructions to move their feet in any particular way or explicitly walk faster. Individuals with HD may have focused mainly on synchronization without lengthening their stride; thus, velocity was reduced relative to PRE.

In the SING tasks, participants were asked to remember the melody and the words of the song and to activate their vocal cords and the appropriate muscles for walking at the same time. This is similar to the DT in that neither singing nor walking was prioritized. We are somewhat surprised that stride length was increased in the SING trials compared to the DT trials. Active music making, including singing, confers several benefits to motor function compared to passive listening [96]. These benefits include increased movement vigor and higher motor output [96,97,98,99]. We speculate that this active music-making element may be a contributing factor to the increases in stride length compared to DT. However, as stride length during SING was still reduced compared to PRE, we do not believe that singing aloud while walking would be beneficial as a gait rehabilitation tool without further research.

We purposely recruited an ambulatory sample of people with HD, with the goal of focusing on people who may benefit from a cueing paradigm. To target this subset of the HD population, we included people who self-reported the ability to walk for ten continuous minutes without assistance. Within this group of relatively mobile participants, we saw varied responsiveness to the cueing tasks, prompting us to further investigate potential contributions to individuals’ ability to walk with a musical cue. While the wide range of values for global cognition, balance confidence, disease severity, and balance ability allowed for appropriate variability to investigate correlations, the limited sample size restricts the interpretation of these exploratory analyses, particularly the non-significant correlations. Past correlation analyses have established that there are many factors that contribute to general gait impairments for individuals with HD, including disease severity and cognition [28,57,83,100]. Greater disease severity, cognitive impairment, and worse balance scores [36] are related to worse gait impairment, including greater variability and slower velocity at the preferred walking speed [26,36,57]. This includes the cohort herein, as Tueth et al. found significant correlations between uncued walking velocity and the BESTest, UHDRS-TMS, and MoCA [36]. Tueth et al.’s results indicated that individuals with worse balance, worse disease severity, and worse cognition walked slower during comfortable pace walking [36]. To our knowledge, only Thaut et al. looked at potential differences in responsiveness to cues based on other characteristics, and no statistics were run [26]. Grouping individuals by disability, those with the highest level of disability were the least able to entrain their steps or increase their gait velocity while walking to a metronome or musical cue [26]. In our exploratory analyses, associations were similar between cueing types. There were significant associations between the percent change in cadence during cueing and global cognition, as measured with the MoCA, and perceived balance confidence, as measured with the ABC. There was no significant association for disease severity, as measured with the UHDRS-TMS, or balance ability, as measured via the BESTest.

For balance confidence, individuals who demonstrated worse balance confidence were more likely to reduce their cadence, rather than increase their cadence, during cued tasks. Although the correlation was in the same general direction, balance ability, as measured by the BESTest, was not significantly correlated with cadence change, demonstrating that individual perception of balance was more related to cadence change than was actual balance ability. Tueth et al. reported that neither the ABC and BESTest nor the ABC and UHDRS-TMS were significantly correlated in this sample [36], illustrating a potential mismatch between perception and reality for individuals with HD. Individuals with particularly low balance confidence may have prioritized safety over performance of the task, even if balance ability and motor severity were not particularly burdensome, as illustrated by non-significant correlations with task performance. Interestingly, the ABC was not correlated with the MoCA in this sample [36], illustrating that general cognition is not a particularly influential factor on balance confidence. As individuals with HD may have limited insight into their balance ability [13,101], balance confidence may be of particular interest for future investigations of rehabilitation techniques in those with HD.

Of the four exploratory variables we investigated regarding relatedness to the ability to adjust cadence to a cue, the MoCA demonstrated the strongest correlations. This aligns with the aforementioned research that decreases in cognition are a major contributor to the inability to adapt gait patterns during cueing and other complex gait tasks in HD [26,27,28,29,83,100]. This finding is not exclusive to this population, as individuals with PD and comorbid dementia demonstrate similar inability to adapt to musical cues. In Tueth et al., individuals with a Clinical Dementia Rating score of 0.5–1.0, representing very mild to mild dementia, were not able to significantly increase cadence, stride length, or velocity when either the internal or external cue was set to 110% of baseline cadence [102]. This is similar to the exploratory results found herein, suggesting that cognition may be an important aspect to recognize when utilizing cueing across populations. Of note, of the eight individuals with HD who could be considered to have limited cognitive impairment with MoCA scores greater than 25, there was still a range of responsiveness to both the MUSIC and SING cues (Figure 3a). While repeated practice may be beneficial to improve gait performance during cueing in HD, motor learning is extensively impaired in HD [103,104,105,106], potentially limiting this approach as well. In combination with the results in the present study, we do not recommend external musical cues or internal singing cues to enhance gait in people with HD.

### 4.1. Limitations

A key limitation of this study is the sample size. The incidence of HD is much lower than for other movement disorders, such as PD. Published studies found a prevalence rate for HD of ~4 people per 100,000 [1], compared to the prevalence rate in PD of ~150 per 100,000 [107,108]. This makes recruitment inherently challenging. Our sample of 22 is within the range of sample sizes of prior research investigating the use of cues for individuals with a definite diagnosis of HD. This includes samples of 15 [27], 20 [29], 23 [26], and 30 [28]. Further, our sample is relatively heterogeneous, with a wide range of ages, disease severities, and mobility impairments. While this group represents the range of individuals seeking care in a clinical setting, larger sample sizes would allow for greater statistical power and may provide greater insight into why some individuals were able to respond to the cues while others were not. Future studies may want to recruit specific sub-populations of individuals with HD to analyze the effects of musical cues on more homogenous groups. Furthermore, while we attempted to control statistical error through appropriate statistical corrections for multiple comparisons, the number of pairwise comparisons combined with the smaller sample is an additional limitation of this study. As such, the results should be interpreted with caution. Although the present study did not find musical cueing useful for improving gait in HD at the group level, future studies may want to investigate the appropriateness of music-based cueing on an individual level.

We chose our methods to align with the previous research, but these choices may have impacted our results. First, we purposefully did not randomize the trial order so that all individuals had the same time to integrate the singing cue. While this was performed in an effort to improve learning and performance of the novel singing task, the fixed order of the walking conditions may have introduced aspects of bias that we could not control, including fatigue effects. Similar to Thaut et al., we compared comfortable pace walking before and after the cueing tasks [26]. While Thaut and colleagues found a significant increase in gait velocity [26], we found no significant difference between PRE and POST trials. Indeed, in our sample, individuals walked with nearly identical cadence, speed, and stride length during PRE and POST (Appendix C). While we did not find a carryover effect, individuals did not walk significantly slower after the trials, suggesting that walking fatigue may not be a substantial factor in their ability to complete the task. Future studies should investigate whether trial order impacts individuals’ ability to utilize the cues appropriately.

Second, we chose to have all participants walk at 110% of PRE cadence and had all participants walk to the same song. While there was an aspect of personalization, as tempo was set as a percentage of individual baseline cadence, further individualization of the cue may have elicited greater benefits. For example, our previous work in people with PD demonstrated that not all participants respond similarly to the same cueing percentage [44]. In Harrison et al., 2025 [44], more than 60% of participants had the longest strides at 120% of baseline walking, but there were individuals who demonstrated the longest stride at 90%, 100%, and 110%. While our sample herein was not able to improve gait as a group in response to either cue, participants differed in cognitive ability, balance ability, and disease severity. In the future, it may be fruitful to determine optimal tempo on an individual basis. Also, in the interest of simplicity, we only used one song across all trials. This was done to control the effect of musical variation. Variation in genre [109], beat salience [110], and groove [111] may influence the success of cueing paradigms. We also did not measure enjoyment of the song in our participants, which may be a factor that influences movement vigor [112]. These aspects should be considered in future studies.

Another limitation was that we did not analyze synchronization or determine the success of each participant’s ability to complete either task. We did not have time-synchronized walking and auditory data, limiting our ability to explore synchronization or entrainment in this sample. This would be of particular interest in future studies to investigate how close or far participants were from being “on beat” and how secondary variables of stride length, speed, and variability may be affected by how synchronized participants are. Regarding people with PD, Harrison and colleagues noted that all participants were able to do the SING tasks with “apparent ease.” This was not the case for our sample of individuals with HD. While all individuals were able to sing the song seated, many struggled to remember the lyrics or the overall melody of the song while walking. As the MUSIC trials were not significantly different from the SING trials, the specific task of singing the song aloud was clearly not the only factor impacting performance. No participants commented on either cueing task being hard or challenging. However, participants were not asked any questions on usability or difficulty directly. Investigating participants’ perceptions of the task would provide important insight into how effective cues may be in a rehabilitation setting.

In the current paper, we consider greater variability in stride length and stride time to be an indicator of timing dysfunction [57] and instability [76,77,78,79,83]. There is the potential that greater variability may represent greater exploration [113] during locomotion and may not necessarily be a negative aspect of gait [114]. As cueing tasks may be novel to participants, increased variability is expected due to initial learning, but too much variability at baseline is detrimental to the motor learning process [115]. As gait variability is higher in those with HD compared to those with PD [57], the amount of baseline variability may be too great to utilize the cues appropriately. In the present sample, the significant differences observed in variability during cueing exceeded the change in variability observed during DT, a phenomenon not observed in people with PD [42]. Investigating changes in gait variability with walking and gait rehabilitation would be helpful to discern the effects of gait variability on mobility impairment in those with HD.

### 4.2. Clinical Implications

Due to the several limitations of this study, we are cautious to make recommendations for clinicians in their use of cueing as a rehabilitation tool. Our data suggest that, as a group, the 22 ambulatory individuals with HD could not, or did not, improve their walking performance with cues. Importantly, we did not observe any adverse events or falls during the cueing tasks. However, gait variability did increase, potentially indicating unsteady movement patterns. The safety and efficacy of cueing as a long-term gait rehabilitation tool have not been assessed in this population. New and challenging mobility tasks should always be performed under the supervision of professionals to avoid injuries. As shown with the exploratory correlation analysis, there were a few individuals who were able to increase their cadence during the cueing task. While we did not conduct a “responder” analysis to expand on our correlations, this type of analysis may be of relevance in future larger studies [116,117]. If a clinician thinks that music-based cueing may be beneficial for an individual patient, it may be useful to consider the individual’s cognitive ability and perceptions of their balance.

## 5. Conclusions

This study represents the first direct comparison between a cognitive DT and external and internal rhythmic auditory cueing in individuals with HD. It is also the first study to investigate the relationship between key characteristics related to mobility and individuals’ ability to respond to rhythmic auditory cueing. Our results suggest that externally played and internally generated musical cues do not significantly improve gait and may even increase gait variability, potentially worsening gait stability. The cueing tasks were somewhat similar to the cognitive DT, as both the DT and cueing trials increased variability compared to the baseline. Greater subjective balance confidence and global cognitive performance were associated with greater ability to change cadence during cueing tasks, suggesting the high cognitive demand of cues may be a limitation for individuals with HD. Based on our findings, we advise caution when using music-based cues to enhance gait in HD.

## Figures and Tables

**Table 1:** Demographics.

Variable	Mean ± SD	Range
Age (years)	54 ± 12	[31–74]
Age of Symptom Onset (years)	44 ± 10	[28–61]
Male, Female (count)	*n* = 10, *n* = 12	NA
Falls in the Previous Month	1 ± 2	[0–10]
MoCA (points)	22 ± 5	[10–29]
UHDRS-TMS (points)	22 ± 16	[2–54]
BESTest (points)	72 ± 19	[42–101]
ABC (%)	75% ± 25%	[23–100]

Abbreviations: standard deviation (SD); Montreal Cognitive Assessment (MoCA); Unified Huntington’s Disease Rating Scale Total Motor Score (UHDRS-TMS); Balance Evaluation Systems Test (BESTest); and Activities-Specific Balance Confidence Scale (ABC).

**Table 2:** Means and standard deviations for gait variables across all four tasks.

Gait Variable	PRE	DT	MUSIC	SING
Cadence (steps/min)	109.75 ± 11.52	104.06 ± 12.97	111.22 ± 13.7	106.48 ± 14.61
Stride length (m)	1.19 ± 0.19	1.11 ± 0.17	1.13 ± 0.24	1.18 ± 0.22
Velocity (m/s)	1.08 ± 0.2	0.96 ± 0.17	1.06 ± 0.27	1.05 ± 0.26
Stride length CV (%)	2.58 ± 2.28	3.30 ± 2.43	3.73 ± 3.28	3.04 ± 2.31
Stride time CV (%)	1.85 ± 1.07	2.98 ± 2.21	2.60 ± 1.54	2.54 ± 1.64

Abbreviations: baseline, uncued walking trials (PRE); cognitive dual-task trials (DT); externally based cue of music being played aloud (MUSIC); internally generated cueing of participants singing aloud (SING); and coefficient of variation (CV).

## Data Availability

The data presented in this study are available upon reasonable request from the corresponding author.

## Abbreviations

The following abbreviations are used in this manuscript:

HD	Huntington’s disease
PD	Parkinson’s disease
RAS	Rhythmic auditory stimulation
MoCA	Montreal Cognitive Assessment
UHDRS-TMS	Unified Huntington’s Disease Rating Scale Total Motor Score
BESTest	Balance Evaluation Systems Test
MUSIC	externally based cue of music being played aloud
SING	internally generated cueing of participants singing aloud
PRE	baseline, uncued walking trials
DT	cognitive dual-task trials
POST	final, uncued walking trials
CV	coefficient of variation
ABC	Activities-Specific Balance Confidence Scale
T-MoCA	Telephone MoCA

## Appendix A

**Table A1:** Participant specific demographics and medication status.

ID	Age	Age of Onset	Gender	Falls in the Last Month	MoCA	UHDRS-TMS	BESTest	ABC	VMATInhibitor	Benzo-Diazepine	Anti-Psychotic	Tetracyclic Antidepressant	SNRI	SSRI	Anticonvulsant
1	64	41	male	3	11	33	42	30	N	Y	Y	Y	Y	N	N
2	63	55	female	0	21	27	65	100	N	Y	N	Y	N	Y	N
3	31	28	female	2	25	3	101	95	N	N	N	N	N	Y	N
4	65	61	male	0	18	24	63	100	N	N	N	N	N	N	N
5	48	41	male	0	28	4	96	100	N	N	N	N	N	N	N
6	74	59	male	0	20	38	53	66.9	N	N	N	N	N	Y	Y
7	43	33	female	0	23	13	78	91.3	N	N	Y	Y	N	Y	Y
8	70	48	male	0	25	24	67	53.8	N	N	Y	N	N	Y	N
9	61	50	female	0	20	47	46	71.3	N	N	Y	N	N	Y	N
10	53	46	male	6	19	46	47	35	N	Y	Y	Y	N	Y	Y
11	56	43	male	0	26	31	70	92.5	N	N	N	N	N	N	N
12	65	54	male	0	21	54	51	93.8	Y	N	N	N	N	Y	N
13	54	35	female	0	28	17	83	93.1	N	N	N	N	N	N	N
14	68	59	female	0	17	45	44	76.3	N	N	Y	Y	N	Y	N
15	62	52	male	0	16	32	61	23.1	Y	N	N	N	N	Y	Y
16	37	36	female	0	29	3	95	91.9	N	N	N	N	N	N	N
17	34	29	female	0	22	16	73	51.9	N	Y	N	Y	Y	N	N
18	41	32	female	2	26	2	96	77.5	N	N	N	N	N	N	N
19	57	33	female	10	22	8	86	51.3	N	N	N	N	N	Y	N
20	45	42	female	1	28	17	83	76.3	N	Y	N	N	N	Y	N
21	50	46	female	0	26	5	79	91.3	N	N	N	N	Y	N	N
22	46	34	male	1	26	8	97	99.4	N	N	N	N	N	N	N

Abbreviations: Montreal Cognitive Assessment (MoCA); Unified Huntington’s Disease Rating Scale Total Motor Score (UHDRS-TMS); Balance Evaluation Systems Test (BESTest); Activities-Specific Balance Confidence Scale (ABC); vesicular monoamine transporter (VMAT); serotonin-norepinephrine reuptake inhibitor (SNRI); selective serotonin reuptake inhibitor (SSRI); Yes, the individual was taking regular medication in this drug class (Y); No, the individual was not taking medication in this drug class (N).

## Appendix B

Both log-transformed variability measures are shown in Table A2.

**Table A2:** Log-transformed measures of variability.

Gait Variable	PRE	DT	MUSIC	SING
Log (stride length CV)	−1.67 ± 0.25	−1.56 ± 0.26	−1.53 ± 0.27	−1.60 ± 0.26
Log (stride time CV)	−1.79 ± 0.21	−1.60 ± 0.25	−1.65 ± 0.23	−1.67 ± 0.26

Abbreviations: baseline, uncued walking trials (PRE); cognitive dual-task trials (DT); externally based cue of music being played aloud (MUSIC); internally generated cueing of participants singing aloud (SING); and coefficient of variation (CV).

## Appendix C

No measures of gait were significantly different between PRE and POST (Table A3).

**Table A3:** Gait variables of participants at PRE and POST.

Gait Variable	PRE	POST	t(df) = t, *p*
Cadence (steps/min)	109.75 ± 11.52	109.04 ± 12.51	t(21) = 0.630, *p* = 0.535
Stridelength (m)	1.19 ± 0.19	1.19 ± 0.19	t(21) = 0.019, *p* = 0.985
Velocity (m/s)	1.08 ± 0.20	1.08 ± 0.21	t(21) = 0.229, *p* = 0.821
Stride length CV (%) *	2.58 ± 2.28	2.53 ± 2.00	t(21) = 0.352, *p* = 0.729
Stride time CV (%) *	1.85 ± 1.07	1.98 ± 1.15	t(21) = −0.866, *p* = 0.396
Log (stride length CV) *	−1.67 ± 0.25	−1.68 ± 0.26	t(21) = 0.321, *p* = 0.752
Log (stride time CV) *	−1.79 ± 0.21	−1.77 ± 0.25	t(21) = −0.530, *p* = 0.601

* Variability in stride length and stride time was not normally distributed. After log-transformation, the variables were normally distributed. Both original and log-transformed data and statistics are presented herein. Abbreviations: baseline, uncued walking trials (PRE); final, uncued walking trials (POST); and coefficient of variation (CV).

## References

[1] Medina A., Mahjoub Y., Shaver L., Pringsheim T. (2022). Prevalence and Incidence of Huntington’s Disease: An Updated Systematic Review and Meta-Analysis. Mov. Disord..

[2] Overview of Huntington’s Disease. https://hdsa.org/what-is-hd/overview-of-huntingtons-disease.

[3] Shannon K.M. (2011). Huntington’s Disease—Clinical Signs, Symptoms, Presymptomatic Diagnosis, and Diagnosis. Handbook of Clinical Neurology.

[4] Girotti F., Marano R., Soliveri P., Geminiani G., Scigliano G. (1988). Relationship between Motor and Cognitive Disorders in Huntington’s Disease. J. Neurol..

[5] Bradshaw J.L., Phillips J.G., Dennis C., Mattingley J.B., Andrewes D., Chiu E., Pierson J.M., Bradshaw J.A. (1992). Initiation and Execution of Movement Sequences in Those Suffering from and At-Risk of Developing Huntington’s Disease. J. Clin. Exp. Neuropsychol..

[6] Rao A.K., Muratori L., Louis E.D., Moskowitz C.B., Marder K.S. (2008). Spectrum of Gait Impairments in Presymptomatic and Symptomatic Huntington’s Disease. Mov. Disord..

[7] Kirkwood S.C., Su J.L., Conneally P.M., Foroud T. (2001). Progression of Symptoms in the Early and Middle Stages of Huntington Disease. Arch. Neurol..

[8] Grimbergen Y.A., Munneke M., Bloem B.R. (2004). Falls in Parkinson’s Disease. Curr. Opin. Neurol..

[9] Delval A., Krystkowiak P., Blatt J.-L., Labyt E., Dujardin K., Destée A., Derambure P., Defebvre L. (2006). Role of Hypokinesia and Bradykinesia in Gait Disturbances in Huntington’s Disease: A Biomechanical Study. J. Neurol..

[10] Vuong K., Canning C.G., Menant J.C., Loy C.T. (2018). Gait, Balance, and Falls in Huntington Disease. Handbook of Clinical Neurology.

[11] Dalton A., Khalil H., Busse M., Rosser A., Van Deursen R., ÓLaighin G. (2013). Analysis of Gait and Balance through a Single Triaxial Accelerometer in Presymptomatic and Symptomatic Huntington’s Disease. Gait Posture.

[12] Gaßner H., Jensen D., Marxreiter F., Kletsch A., Bohlen S., Schubert R., Muratori L.M., Eskofier B., Klucken J., Winkler J. (2020). Gait Variability as Digital Biomarker of Disease Severity in Huntington’s Disease. J. Neurol..

[13] Grimbergen Y.A.M., Knol M.J., Bloem B.R., Kremer B.P.H., Roos R.A.C., Munneke M. (2008). Falls and Gait Disturbances in Huntington’s Disease. Mov. Disord..

[14] Quinn L., Rao A. (2002). Physical Therapy for People with Huntington Disease: Current Perspectives and Case Report. J. Neurol. Phys. Ther..

[15] Moon Y., Sung J., An R., Hernandez M.E., Sosnoff J.J. (2016). Gait Variability in People with Neurological Disorders: A Systematic Review and Meta-Analysis. Hum. Mov. Sci..

[16] Tyagi S., Shekhar N., Thakur A.K. (2024). Alternative Approaches for the Management of Huntington’s Disease: A Narrative Review. Altern. Ther. Health Med..

[17] Travessa A.M., Rodrigues F.B., Mestre T.A., Ferreira J.J. (2017). Fifteen Years of Clinical Trials in Huntington’s Disease: A Very Low Clinical Drug Development Success Rate. J. Huntingt. Dis..

[18] Ferreira J.J., Rodrigues F.B., Duarte G.S., Mestre T.A., Bachoud-Levi A., Bentivoglio A.R., Burgunder J., Cardoso F., Claassen D.O., Landwehrmeyer G.B. (2022). An MDS Evidence-Based Review on Treatments for Huntington’s Disease. Mov. Disord..

[19] Saft C., Burgunder J.-M., Dose M., Jung H.H., Katzenschlager R., Priller J., Nguyen H.P., Reetz K., Reilmann R., Seppi K. (2023). Symptomatic Treatment Options for Huntington’s Disease (Guidelines of the German Neurological Society). Neurol. Res. Pr..

[20] Quinn L., Kegelmeyer D., Kloos A., Rao A.K., Busse M., Fritz N.E. (2020). Clinical Recommendations to Guide Physical Therapy Practice for Huntington Disease. Neurology.

[21] Thaut M.H., Abiru M. (2010). Rhythmic Auditory Stimulation in Rehabilitation of Movement Disorders: A Review of Current Research. Music. Percept..

[22] Nieuwboer A., Kwakkel G., Rochester L., Jones D., van Wegen E., Willems A.M., Chavret F., Hetherington V., Baker K., Lim I. (2007). Cueing Training in the Home Improves Gait-Related Mobility in Parkinson’s Disease: The RESCUE Trial. J. Neurol. Neurosurg. Psychiatry.

[23] Muthukrishnan N., Abbas J.J., Shill H.A., Krishnamurthi N. (2019). Cueing Paradigms to Improve Gait and Posture in Parkinson’s Disease: A Narrative Review. Sensors.

[24] Spaulding S.J., Barber B., Colby M., Cormack B., Mick T., Jenkins M.E. (2013). Cueing and Gait Improvement Among People with Parkinson’s Disease: A Meta-Analysis. Arch. Phys. Med. Rehabil..

[25] Harrison E.C., Earhart G.M. (2023). The Effect of Auditory Cues on Gait Variability in People with Parkinson’s Disease and Older Adults: A Systematic Review. Neurodegener. Dis. Manag..

[26] Thaut M.H., Miltner R., Lange H.W., Hurt C.P., Hoemberg V. (1999). Velocity Modulation and Rhythmic Synchronization of Gait in Huntington’s Disease. Mov. Disord..

[27] Delval A., Krystkowiak P., Delliaux M., Blatt J., Derambure P., Destée A., Defebvre L. (2008). Effect of External Cueing on Gait in Huntington’s Disease. Mov. Disord..

[28] Bilney B., Morris M.E., Churchyard A., Chiu E., Georgiou-Karistianis N. (2005). Evidence for a Disorder of Locomotor Timing in Huntington’s Disease. Mov. Disord..

[29] Churchyard A., Morris M., Georgiou-Karistianis N., Chiu E., Cooper R., Iansek R. (2001). Gait Dysfunction in Huntington’s Disease: Parkinsonism and a Disorder of Timing. Implic. Mov. Rehabil. Adv. Neurol..

[30] Kim K.-H., Song M.-K. (2023). Update of Rehabilitation in Huntington’s Disease: Narrative Review. Brain Neurorehabil..

[31] Schwartz A.E., Van Walsem M.R., Brean A., Frich J.C. (2019). Therapeutic Use of Music, Dance, and Rhythmic Auditory Cueing for Patients with Huntington’s Disease: A Systematic Review. J. Huntingt. Dis..

[32] De Dreu M.J., Van Der Wilk A.S.D., Poppe E., Kwakkel G., Van Wegen E.E.H. (2012). Rehabilitation, Exercise Therapy and Music in Patients with Parkinson’s Disease: A Meta-Analysis of the Effects of Music-Based Movement Therapy on Walking Ability, Balance and Quality of Life. Park. Relat. Disord..

[33] Rodger M.W.M., Craig C.M. (2016). Beyond the Metronome: Auditory Events and Music May Afford More than Just Interval Durations as Gait Cues in Parkinson’s Disease. Front. Neurosci..

[34] Rose D., Delevoye-Turrell Y., Ott L., Annett L.E., Lovatt P.J. (2019). Music and Metronomes Differentially Impact Motor Timing in People with and without Parkinson’s Disease: Effects of Slow, Medium, and Fast Tempi on Entrainment and Synchronization Performances in Finger Tapping, Toe Tapping, and Stepping on the Spot Tasks. Park. Dis..

[35] Harrison E.C., Horin A.P., Earhart G.M. (2018). Internal Cueing Improves Gait More than External Cueing in Healthy Adults and People with Parkinson Disease. Sci. Rep..

[36] Tueth L.E., Haussler A.M., Baudendistel S.T., Earhart G.M. (2024). Exploring Relationships among Gait, Balance, and Physical Activity in Individuals with Huntington’s Disease. J. Huntington’s Dis..

[37] Nasreddine Z.S., Phillips N.A., Bedirian V., Charbonneau S., Whitehead V., Collin I., Cummings J.L., Chertkow H. (2005). The Montreal Cognitive Assessment, MoCA: A Brief Screening Tool for Mild Cognitive Impairment. J. Am. Geriatr. Soc..

[38] Bezdicek O., Majerova V., Novak M., Nikolai T., Ruzicka E., Roth J. (2013). Validity of the Montreal Cognitive Assessment in the Detection of Cognitive Dysfunction in Huntington’s Disease. Appl. Neuropsychol. Adult.

[39] Mestre T.A., Forjaz M.J., Mahlknecht P., Cardoso F., Ferreira J.J., Reilmann R., Sampaio C., Goetz C.G., Cubo E., Martinez-Martin P. (2018). Rating Scales for Motor Symptoms and Signs in Huntington’s Disease: Critique and Recommendations. Mov. Disord. Clin. Pr..

[40] Horak F.B., Wrisley D.M., Frank J. (2009). The Balance Evaluation Systems Test (BESTest) to Differentiate Balance Deficits. Phys. Ther..

[41] Mancini M., King L., Salarian A., Holmstrom L., McNames J., Horak F.B. (2011). Mobility Lab to Assess Balance and Gait with Synchronized Body-Worn Sensors. J. Bioeng. Biomed. Sci..

[42] Harrison E.C., McNeely M.E., Earhart G.M. (2017). The Feasibility of Singing to Improve Gait in Parkinson Disease. Gait Posture.

[43] Park K.S., Hass C.J., Janelle C.M. (2021). Familiarity with Music Influences Stride Amplitude and Variability during Rhythmically-Cued Walking in Individuals with Parkinson’s Disease. Gait Posture.

[44] Harrison E.C., Tueth L.E., Haussler A.M., Rawson K.S., Earhart G.M. (2025). Personalized Auditory Rhythmic Cues to Optimize Gait in Older Adults and People with Parkinson Disease. J. Neurol. Phys. Ther..

[45] Browning S., Holland S., Wellwood I., Bilney B. (2023). Spatiotemporal Gait Parameters in Adults with Premanifest and Manifest Huntington’s Disease: A Systematic Review. J. Mov. Disord..

[46] Powell L.E., Myers A.M. (1995). The Activities-Specific Balance Confidence (ABC) Scale. J. Gerontol. Ser. A Biol. Sci. Med. Sci..

[47] Stasny B.M., Newton R.A., Viggiano LoCascio L., Bedio N., Lauke C., Conroy M., Thompson A., Vakhnenko L., Polidoro C. (2011). The ABC Scale and Fall Risk: A Systematic Review. Phys. Occup. Ther. Geriatr..

[48] Lord S., Galna B., Verghese J., Coleman S., Burn D., Rochester L. (2013). Independent Domains of Gait in Older Adults and Associated Motor and Nonmotor Attributes: Validation of a Factor Analysis Approach. J. Gerontol. Ser. A Biol. Sci. Med. Sci..

[49] Katz M.J., Wang C., Nester C.O., Derby C.A., Zimmerman M.E., Lipton R.B., Sliwinski M.J., Rabin L.A. (2021). T-MoCA: A Valid Phone Screen for Cognitive Impairment in Diverse Community Samples. Alzheimer’s Dement. Diagn. Assess. Dis. Monit..

[50] Ghai S., Ghai I., Schmitz G., Effenberg A.O. (2018). Effect of Rhythmic Auditory Cueing on Parkinsonian Gait: A Systematic Review and Meta-Analysis. Sci. Rep..

[51] Wittwer J.E., Webster K.E., Hill K. (2013). Rhythmic Auditory Cueing to Improve Walking in Patients with Neurological Conditions Other than Parkinson’s Disease—What Is the Evidence?. Disabil. Rehabil..

[52] Alexi T. (2000). Neuroprotective Strategies for Basal Ganglia Degeneration: Parkinson’s and Huntington’s Diseases. Prog. Neurobiol..

[53] Aylward E.H., Li Q., Stine O.C., Ranen N., Sherr M., Barta P.E., Bylsma F.W., Pearlson G.D., Ross C.A. (1997). Longitudinal Change in Basal Ganglia Volume in Patients with Huntington’s Disease. Neurology.

[54] Takakusaki K. (2013). Neurophysiology of Gait: From the Spinal Cord to the Frontal Lobe. Mov. Disord..

[55] Takakusaki K., Tomita N., Yano M. (2008). Substrates for Normal Gait and Pathophysiology of Gait Disturbances with Respect to the Basal Ganglia Dysfunction. J. Neurol..

[56] Mirelman A., Bonato P., Camicioli R., Ellis T.D., Giladi N., Hamilton J.L., Hass C.J., Hausdorff J.M., Pelosin E., Almeida Q.J. (2019). Gait Impairments in Parkinson’s Disease. Lancet Neurol..

[57] Hausdorff J.M., Cudkowicz M.E., Firtion R., Wei J.Y., Goldberger A.L. (1998). Gait Variability and Basal Ganglia Disorders: Stride-to-Stride Variations of Gait Cycle Timing in Parkinson’s Disease and Huntington’s Disease. Mov. Disord..

[58] Harrison E.C., Grossen S., Tueth L.E., Haussler A.M., Rawson K.S., Campbell M.C., Earhart G.M. (2025). Neural Mechanisms Underlying Synchronization of Movement to Musical Cues in Parkinson Disease and Aging. Front. Neurosci..

[59] Bengtsson S.L., Ullén F., Henrik Ehrsson H., Hashimoto T., Kito T., Naito E., Forssberg H., Sadato N. (2009). Listening to Rhythms Activates Motor and Premotor Cortices. Cortex.

[60] Chen J.L., Zatorre R.J., Penhune V.B. (2006). Interactions between Auditory and Dorsal Premotor Cortex during Synchronization to Musical Rhythms. NeuroImage.

[61] Grahn J.A., Brett M. (2007). Rhythm and Beat Perception in Motor Areas of the Brain. J. Cogn. Neurosci..

[62] Bijsterbosch J.D., Lee K.-H., Hunter M.D., Tsoi D.T., Lankappa S., Wilkinson I.D., Barker A.T., Woodruff P.W.R. (2011). The Role of the Cerebellum in Sub- and Supraliminal Error Correction during Sensorimotor Synchronization: Evidence from fMRI and TMS. J. Cogn. Neurosci..

[63] Thaut M.H., Stephan K.M., Wunderlich G., Schicks W., Tellmann L., Herzog H., McIntosh G.C., Seitz R.J., Hömberg V. (2009). Distinct Cortico-Cerebellar Activations in Rhythmic Auditory Motor Synchronization. Cortex.

[64] Martinu K., Monchi O. (2013). Cortico-Basal Ganglia and Cortico-Cerebellar Circuits in Parkinson’s Disease: Pathophysiology or Compensation?. Behav. Neurosci..

[65] Torres E.B., Heilman K.M., Poizner H. (2011). Impaired Endogenously Evoked Automated Reaching in Parkinson’s Disease. J. Neurosci..

[66] Mirdamadi J.L. (2016). Cerebellar Role in Parkinson’s Disease. J. Neurophysiol..

[67] Tereshchenko A.V., Schultz J.L., Bruss J.E., Magnotta V.A., Epping E.A., Nopoulos P.C. (2020). Abnormal Development of Cerebellar-Striatal Circuitry in Huntington Disease. Neurology.

[68] Feigin A., Tang C., Ma Y., Mattis P., Zgaljardic D., Guttman M., Paulsen J.S., Dhawan V., Eidelberg D. (2007). Thalamic Metabolism and Symptom Onset in Preclinical Huntington’s Disease. Brain.

[69] Yu H., Sternad D., Corcos D.M., Vaillancourt D.E. (2007). Role of Hyperactive Cerebellum and Motor Cortex in Parkinson’s Disease. NeuroImage.

[70] Rüb U., Hoche F., Brunt E.R., Heinsen H., Seidel K., Del Turco D., Paulson H.L., Bohl J., Von Gall C., Vonsattel J. (2013). Degeneration of the Cerebellum in Huntington’s Disease (HD): Possible Relevance for the Clinical Picture and Potential Gateway to Pathological Mechanisms of the Disease Process. Brain Pathol..

[71] Franklin G.L., Camargo C.H.F., Meira A.T., Lima N.S.C., Teive H.A.G. (2021). The Role of the Cerebellum in Huntington’s Disease: A Systematic Review. Cerebellum.

[72] Rees E.M., Farmer R., Cole J.H., Haider S., Durr A., Landwehrmeyer B., Scahill R.I., Tabrizi S.J., Hobbs N.Z. (2014). Cerebellar Abnormalities in Huntington’s Disease: A Role in Motor and Psychiatric Impairment?. Mov. Disord..

[73] Singh-Bains M.K., Mehrabi N.F., Sehji T., Austria M.D., Tan A.Y., Tippett L.J., Dragunow M., Waldvogel H.J., Faull R.L. (2019). Cerebellar Degeneration Correlates with Motor Symptoms in Huntington Disease. Ann. Neurol..

[74] Solstrand Dahlberg L., Lungu O., Doyon J. (2020). Cerebellar Contribution to Motor and Non-Motor Functions in Parkinson’s Disease: A Meta-Analysis of fMRI Findings. Front. Neurol..

[75] Hannaway N., Lao-Kaim N.P., Martín-Bastida A., Roussakis A.-A., Howard J., Wall M.B., Loane C., Barker R.A., Piccini P. (2021). Longitudinal Changes in Movement-Related Functional MRI Activity in Parkinson’s Disease Patients. Park. Relat. Disord..

[76] Hausdorff J.M. (2009). Gait Dynamics in Parkinson’s Disease: Common and Distinct Behavior among Stride Length, Gait Variability, and Fractal-like Scaling. Chaos.

[77] Brach J.S., Berlin J.E., VanSwearingen J.M., Newman A.B., Studenski S.A. (2005). Too Much or Too Little Step Width Variability Is Associated with a Fall History in Older Persons Who Walk at or near Normal Gait Speed. J. Neuroeng. Rehabil..

[78] Brach J.S., Studenski S., Perera S., VanSwearingen J.M., Newman A.B. (2008). Stance Time and Step Width Variability Have Unique Contributing Impairments in Older Persons. Gait Posture.

[79] Beauchet O., Allali G., Annweiler C., Bridenbaugh S., Assal F., Kressig R.W., Herrmann F.R. (2009). Gait Variability among Healthy Adults: Low and High Stride-to-Stride Variability Are Both a Reflection of Gait Stability. Gerontology.

[80] Muratori L.M., Quinn L., Li X., Youdan G., Busse M., Fritz N.E. (2021). Measures of Postural Control and Mobility during Dual-Tasking as Candidate Markers of Instability in Huntington’s Disease. Hum. Mov. Sci..

[81] Purcell N.L., Goldman J.G., Ouyang B., Liu Y., Bernard B., O’Keefe J.A. (2020). The Effects of Dual-Task Cognitive Interference on Gait and Turning in Huntington’s Disease. PLoS ONE.

[82] Fritz N.E., Hamana K., Kelson M., Rosser A., Busse M., Quinn L. (2016). Motor-Cognitive Dual-Task Deficits in Individuals with Early-Mid Stage Huntington Disease. Gait Posture.

[83] Delval A., Krystkowiak P., Delliaux M., Dujardin K., Blatt J., Destée A., Derambure P., Defebvre L. (2008). Role of Attentional Resources on Gait Performance in Huntington’s Disease. Mov. Disord..

[84] Radovanović S., Vodopić S., Stanković I., Dragašević-Mišković N., Kostić V. (2020). Spatiotemporal Gait Characteristics of Huntington’s Disease during Dual-Task Walking. Int. J. Neurosci..

[85] Kloos A.D., Kegelmeyer D.A., Fritz N.E., Daley A.M., Young G.S., Kostyk S.K. (2017). Cognitive Dysfunction Contributes to Mobility Impairments in Huntington’s Disease. J. Huntington’s Dis..

[86] Vaportzis E., Georgiou-Karistianis N., Churchyard A., Stout J.C. (2015). Dual Task Performance May Be a Better Measure of Cognitive Processing in Huntington’s Disease than Traditional Attention Tests. J. Huntingt. Dis..

[87] Sprengelmeyer R., Lange H., Hömberg V. (1995). The Pattern of Attentional Deficits in Huntington’s Disease. Brain.

[88] Georgiou-Karistianis N., Farrow M., Wilson-Ching M., Churchyard A., Bradshaw J.L., Sheppard D.M. (2012). Deficits in Selective Attention in Symptomatic Huntington Disease: Assessment Using an Attentional Blink Paradigm. Cogn. Behav. Neurol..

[89] Aron A.R., Watkins L., Sahakian B.J., Monsell S., Barker R.A., Robbins T.W. (2003). Task-Set Switching Deficits in Early-Stage Huntington’s Disease: Implications for Basal Ganglia Function. J. Cogn. Neurosci..

[90] Migliore S., D’Aurizio G., Curcio G., Squitieri F. (2019). Task-Switching Abilities in Pre-Manifest Huntington’s Disease Subjects. Park. Relat. Disord..

[91] Baker K., Rochester L., Nieuwboer A. (2007). The Immediate Effect of Attentional, Auditory, and a Combined Cue Strategy on Gait During Single and Dual Tasks in Parkinson’s Disease. Arch. Phys. Med. Rehabil..

[92] Lohnes C.A., Earhart G.M. (2011). The Impact of Attentional, Auditory, and Combined Cues on Walking during Single and Cognitive Dual Tasks in Parkinson Disease. Gait Posture.

[93] Rochester L., Rafferty D., Dotchin C., Msuya O., Minde V., Walker R.W. (2010). The Effect of Cueing Therapy on Single and Dual-Task Gait in a Drug Naïve Population of People with Parkinson’s Disease in Northern Tanzania. Mov. Disord..

[94] Rochester L., Nieuwboer A., Baker K., Hetherington V., Willems A.-M., Chavret F., Kwakkel G., Van Wegen E., Lim I., Jones D. (2007). The Attentional Cost of External Rhythmical Cues and Their Impact on Gait in Parkinson’s Disease: Effect of Cue Modality and Task Complexity. J. Neural Transm..

[95] Danoudis M., Iansek R. (2014). Gait in Huntington’s Disease and the Stride Length-Cadence Relationship. BMC Neurol..

[96] Pacchetti C., Mancini F., Aglieri R., Fundarò C., Martignoni E., Nappi G. (2000). Active Music Therapy in Parkinson’s Disease: An Integrative Method for Motor and Emotional Rehabilitation. Psychosom. Med..

[97] Leman M., Moelants D., Varewyck M., Styns F., Van Noorden L., Martens J.-P. (2013). Activating and Relaxing Music Entrains the Speed of Beat Synchronized Walking. PLoS ONE.

[98] Agus T.R., Thorpe S.J., Pressnitzer D. (2010). Rapid Formation of Robust Auditory Memories: Insights from Noise. Neuron.

[99] Dunbar R.I.M., Kaskatis K., MacDonald I., Barra V. (2012). Performance of Music Elevates Pain Threshold and Positive Affect: Implications for the Evolutionary Function of Music. Evol. Psychol..

[100] Cruickshank T., Reyes A., Peñailillo L., Thompson J., Ziman M. (2014). Factors That Contribute to Balance and Mobility Impairments in Individuals with Huntington’s Disease. Basal Ganglia.

[101] Kloos A.D., Fritz N.E., Kostyk S.K., Young G.S., Kegelmeyer D.A. (2014). Clinimetric Properties of the Tinetti Mobility Test, Four Square Step Test, Activities-Specific Balance Confidence Scale, and Spatiotemporal Gait Measures in Individuals with Huntington’s Disease. Gait Posture.

[102] Tueth L.E., Haussler A.M., Lohse K.R., Rawson K.S., Earhart G.M., Harrison E.C. (2024). Effect of Musical Cues on Gait in Individuals with Parkinson Disease with Comorbid Dementia. Gait Posture.

[103] Heindel W.C., Butters N., Salmon D.P. (1988). Impaired Learning of a Motor Skill in Patients with Huntington’s Disease. Behav. Neurosci..

[104] Smith M.A., Shadmehr R. (2005). Intact Ability to Learn Internal Models of Arm Dynamics in Huntington’s Disease But Not Cerebellar Degeneration. J. Neurophysiol..

[105] Smith M.A., Brandt J., Shadmehr R. (2000). Motor Disorder in Huntington’s Disease Begins as a Dysfunction in Error Feedback Control. Nature.

[106] Holtbernd F., Tang C.C., Feigin A., Dhawan V., Ghilardi M.F., Paulsen J.S., Guttman M., Eidelberg D. (2016). Longitudinal Changes in the Motor Learning-Related Brain Activation Response in Presymptomatic Huntington’s Disease. PLoS ONE.

[107] Su D., Cui Y., He C., Yin P., Bai R., Zhu J., Lam J.S.T., Zhang J., Yan R., Zheng X. (2025). Projections for Prevalence of Parkinson’s Disease and Its Driving Factors in 195 Countries and Territories to 2050: Modelling Study of Global Burden of Disease Study 2021. BMJ.

[108] Zhu J., Cui Y., Zhang J., Yan R., Su D., Zhao D., Wang A., Feng T. (2024). Temporal Trends in the Prevalence of Parkinson’s Disease from 1980 to 2023: A Systematic Review and Meta-Analysis. Lancet Healthy Longev..

[109] De Bartolo D., Morone G., Giordani G., Antonucci G., Russo V., Fusco A., Marinozzi F., Bini F., Spitoni G.F., Paolucci S. (2020). Effect of Different Music Genres on Gait Patterns in Parkinson’s Disease. Neurol. Sci..

[110] Park K.S. (2022). Decomposing the Effects of Familiarity with Music Cues on Stride Length and Variability in Persons with Parkinson’s Disease: On the Role of Covariates. IJERPH.

[111] Leow L., Rinchon C., Grahn J. (2015). Familiarity with Music Increases Walking Speed in Rhythmic Auditory Cuing. Ann. New York Acad. Sci..

[112] Mazzoni P., Hristova A., Krakauer J.W. (2007). Why Don’t We Move Faster? Parkinson’s Disease, Movement Vigor, and Implicit Motivation. J. Neurosci..

[113] Newell K.M., McDonald P.V. Searching for Solutions to the Coordination Function: Learning as Exploratory Behavior. In Tutorials in Motor Behavior; North-Holland Publishing, Amsterdam, The Netherlands, 1992.

[114] Harbourne R.T., Stergiou N. (2009). Movement Variability and the Use of Nonlinear Tools: Principles to Guide Physical Therapist Practice. Phys. Ther..

[115] Cardis M., Casadio M., Ranganathan R. (2018). High Variability Impairs Motor Learning Regardless of Whether It Affects Task Performance. J. Neurophysiol..

[116] Harrison E.C., Horin A.P., Myers P.S., Rawson K.S., Earhart G.M. (2020). Changes in Parkinsonian Gait Kinematics with Self-Generated and Externally-Generated Cues: A Comparison of Responders and Non-Responders. Somatosens. Mot. Res..

[117] Baudendistel S.T., Earhart G.M. (2025). Characteristics of Responders to Interventions for Parkinson Disease: A Scoping Systematic Review. Neurodegener. Dis. Manag..

